# Splicing Genomics Events in Cervical Cancer: Insights for Phenotypic Stratification and Biomarker Potency

**DOI:** 10.3390/genes12020130

**Published:** 2021-01-20

**Authors:** Flavia Zita Francies, Sheynaz Bassa, Aristotelis Chatziioannou, Andreas Martin Kaufmann, Zodwa Dlamini

**Affiliations:** 1 SAMRC/UP Precision Prevention & Novel Drug Targets for HIV-Associated Cancers (PPNDTHAC) Extramural Unit, Pan African Cancer Research Institute (PACRI), University of Pretoria, Hatfield 0028, South Africa; flavia.francies@up.ac.za (F.Z.F.); achatzi@bioacademy.gr (A.C.); andreas.kaufmann@charite.de (A.M.K.); 2 Department of Radiation Oncology, Steve Biko Academic Hospital (SBAH), Faculty of Health Sciences, University of Pretoria, Hatfield 0028, South Africa; sheynaz.bassa@up.ac.za; 3 Center of Systems Biology, Biomedical Research Foundation Academy of Athens, 4 Soranou Ephessiou str, 115 27 Athens, Greece; 4 Clinic for Gynaecology, Laboratory for Gynaecologic Tumor Immunology, Institute of Health, Charité-Universitätsmedizin, Freie Universität Berlin, Humboldt-Universität zu Berlin, Augustenburgerplatz 1, 13353 Berlin, Germany

**Keywords:** cervical cancer, alternative splicing, biomarkers, SR proteins, hnRNP, drug resistance

## Abstract

Gynaecological cancers are attributed to the second most diagnosed cancers in women after breast cancer. On a global scale, cervical cancer is the fourth most common cancer and the most common cancer in developing countries with rapidly increasing mortality rates. Human papillomavirus (HPV) infection is a major contributor to the disease. HPV infections cause prominent cellular changes including alternative splicing to drive malignant transformation. A fundamental characteristic attributed to cancer is the dysregulation of cellular transcription. Alternative splicing is regulated by several splicing factors and molecular changes in these factors lead to cancer mechanisms such as tumour development and progression and drug resistance. The serine/arginine-rich (SR) proteins and heterogeneous ribonucleoproteins (hnRNPs) have prominent roles in modulating alternative splicing. Evidence shows molecular alteration and expression levels in these splicing factors in cervical cancer. Furthermore, aberrant splicing events in cancer-related genes lead to chemo- and radioresistance. Identifying clinically relevant modifications in alternative splicing events and splicing variants, in cervical cancer, as potential biomarkers for their role in cancer progression and therapy resistance is scrutinised. This review will focus on the molecular mechanisms underlying the aberrant splicing events in cervical cancer that may serve as potential biomarkers for diagnosis, prognosis, and novel drug targets.

## 1. Introduction

**Figure 1:**
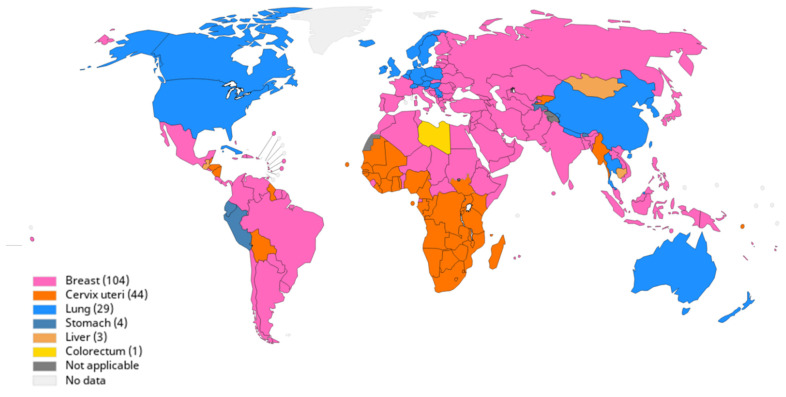
Global cancer mortality. The age standardised rates (ASRs) of various leading cancers worldwide in 2018. Cervical cancer is a major burden in most parts of Africa. Reprinted from [2].

Cervical cancer, also known as cervix uteri cancer, is the fourth most frequently diagnosed cancer globally and the most common malignancy in developing countries [1]. It is the most frequently diagnosed cancer in women in Sub-Saharan Africa (SSA) and the leading cause of cancer-related mortality in this region (Figure 1) [2,3]. An estimated 90% of cervical cancer-related mortality occurs in low- and middle-income countries [4]. Cervical cancer is predominantly categorised into two main histopathological subtypes—squamous cell carcinoma and adenocarcinoma [5]. Over 75–80% of all cervical cancers are squamous cell carcinomas [6,7]. Cervical cancer is attributed to a number of risk factors such as sexually transmitted infections including human immunodeficiency virus (HIV) infection, human papillomavirus (HPV) infection, socioeconomic factors, obesity, smoking [8], alcohol consumption [9], unprotected sex and multiple sexual partners, prolonged usage of oral contraceptives, and family history of cervical cancer [10]. HPV infection is the major contributor of cervical cancer [11]. HPV is a circular double-stranded DNA virus with capsid proteins with more than 200 subtypes identified and categorised as high and low risk. Of these, about 40 subtypes have an affinity for genital mucosa and are sexually transmitted. The low-risk subtypes are generally associated with genital warts, whereas high risk subtypes cause invasive cervical cancer. The most prominent high-risk HPV genotypes are HPV16 and 18. Persistent infection with these high-risk subtypes contributes to over 99% of cervical cancers [11]. HPV infections can be prevented by vaccination that confers protection against HPV 6, 11, 16, and 18 subtypes, and depending on the vaccine, subtypes 31,33,45,52,58 can also be prevented. The vaccinations are available as quadrivalent vaccine to target all four subtypes or as bivalent to target only the high-risk subtypes [12] or a combined 9-valent vaccine that targets nine subtypes [13].

In addition to HPV infections, dysregulated pathways are a fundamental feature in cervical cancer development and progression. For this reason, research in elucidating modifications in cancer-related pathways and alternative splicing is rapidly emerging. Several studies show aberrant alternative splicing and the dysregulation of gene expression in cervical cancer [14,15,16,17]. The related molecular signatures offer potential therapeutic targets for novel drug development and improved strategies in cervical cancer management, particularly for advanced disease in developing countries where HPV infections are the major contributor of cervical cancer. 

The burden of cervical cancer mortality due to HPV infections is felt prominently in developing nations. Novel therapeutic targets are warranted to address this issue. Moreover, prevention strategies such as HPV vaccinations and pap smears play a significant role in cervical cancer prevention. Modifications in cervical tissue are detected through pap smears and HPV tests, and early diagnosis allows effective management of the disease [1,10]. This review will focus on the molecular mechanisms underlying the aberrant splicing events in cervical cancer that may serve as potential biomarkers for diagnosis and prognosis and as novel drug targets for their therapeutic properties.

## 2. Alternative Splicing and Its Implications in Cervical Cancer

**Figure 2:**
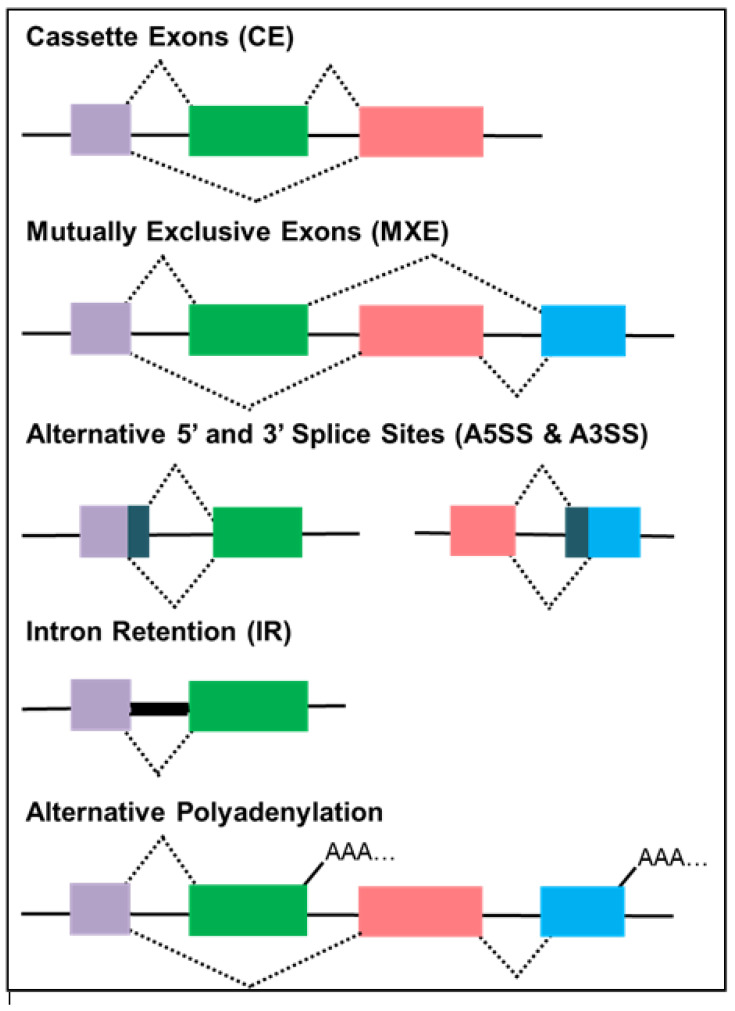
Frequent types of alternative splicing mechanisms. Alternative spliced mRNA produces mature transcripts, namely, cassette exons (CEs), mutually exclusive exons (MXEs), alternative 5′ or 3′ splice sites (A5SS and A3SS), intron retention (IR), and alternative polyadenylation (AP). Coloured boxes: exons; black lines: introns [31,32,33,34,35].

Alternative splicing is an important process in gene expression and proteome diversity. In this cellular process, introns are spliced to join exons for the production of proteins through several mechanisms (Figure 2). Alternative splicing maintains cellular diversity and regulates the synthesis of multiple protein isoforms from the same gene. These protein isoforms perform several biological functions that are necessary for normal cellular functionality. Alternative splicing is an intricate process that is closely regulated by numerous spliceosome factors that aid in recognition of intron and splice sites such as small nuclear ribonucleoproteins (snRNP) particles and the serine/arginine-rich (SR) proteins [18]. In this process, proteins are synthesised, when introns are spliced and functional exons are joined together. The negative regulation of alternative splicing is achieved by heterogeneous ribonucleoproteins (hnRNPs) that block the intron and exon boundaries [19]. These two protein families—the SR proteins and the hnRNPs—are important trans-acting regulatory factors in splicing and are known to be altered in cervical cancer [20,21,22,23]. Enhanced levels of SR lead to splicing induction, whereas splicing is inhibited when hnRNPs are overly expressed. Aberrant alternative splicing, resulting from DNA damage, mutations and expression alterations in splicing factors, miRNA disruptions, and unregulated gene expression, are implicated in cancer mechanisms, such as sustained cell proliferation, apoptotic evasion, tumour suppressor inhibition, angiogenesis, metastasis, and drug resistance [19,24,25,26]. Evidence suggest that aberrant alternative splicing plays an important role in the development of cervical cancer. In cervical cancer, alternative splicing is primarily HPV-mediated. Next generation sequencing (NGS) offers a platform to identify potential disease-causing splice variants and genomic changes in splicing regulatory factors/proteins. Elucidating the functions of these splice variants may provide underlying information on malignant transformation and be beneficial in developing novel strategies for therapeutic interventions [27]. For these reasons, modifications in alternative splicing are becoming a significant biomarker with diagnostic and therapeutic potential.

Alternative splicing of key genes may facilitate the development and progression of cervical malignancy. For instance, the 5′ alternative splicing of the *KLHDC7B* gene is closely associated with cellular differentiation and tumour size in 67.5% of squamous cell carcinoma [28]. Similarly, 35% of exon skipping in the *SYCP2* gene was reported in cervical squamous cell carcinoma and associated with invasion and metastases [28]. Evidence also shows the association of cervical cancer and the aberrant alternative splicing of the *IL1RAP* gene. SRSF10 regulates the splicing of *IL1RAP* gene and promotes the production of its oncogenic isoform, MIL1RAP. This in turn facilitates the malignant cell evasion of phagocytosis by macrophages. Therefore, aberrant alternative splicing of *IL1RAP* gene promotes immune evasion and promotes cervical cancer [29]. A recent study by Ouyang et al. (2020) provides evidence that supports the notion that aberrant splicing events are closely associated with cervical cancer development, and the identification of these splicing biomarkers may provide useful prognostic and therapeutic tools [30]. The authors identified 2860 alternative splicing events. Of these, SNRPA and CCDC12 were associated with the tumour suppressor gene, p53, and were identified as hub genes in cervical cancer [30]. These results highlight the need to screen candidate biomarkers associated with cervical cancer that may have a clinical utility in diagnosis, prognosis, and therapy. Biomarkers related to cervical cancer are shown in Table 1.

HPV contributes to the development and progression of cervical cancer by disrupting alternative splicing and other cellular functions. The HPV genome is double-stranded and circular; it is divided into three regions, namely, the long control region (LCR) and early and late region. Each region produces proteins that have different functions in the life cycle of HPV and in cancer development [36]. Persistent HPV infection gives rise to malignancy by producing viral proteins necessary to maintain virus replication and oncoproteins. Viral oncoproteins facilitate disease development and progression by abrogating normal cellular functions such as G1 arrest, cell proliferation, apoptosis, DNA repair, and chromosomal instability [37]. In addition, HPV oncoproteins bind to splicing factors and induce aberrant alternative splicing events. Moreover, HPV-related cervical cancer has a number of genes and splicing factors that are significantly upregulated. These include genes with vital functions such as immune surveillance, inflammatory response, and tumour suppressors [29,37]. Collectively, the interference of HPV in alternative splicing and cellular function promotes transformation, leading to carcinogenesis.

### 2.1. HPV-Mediated Disruptions in Serine/Arginine-Rich (SR) Proteins

**Figure 3:**
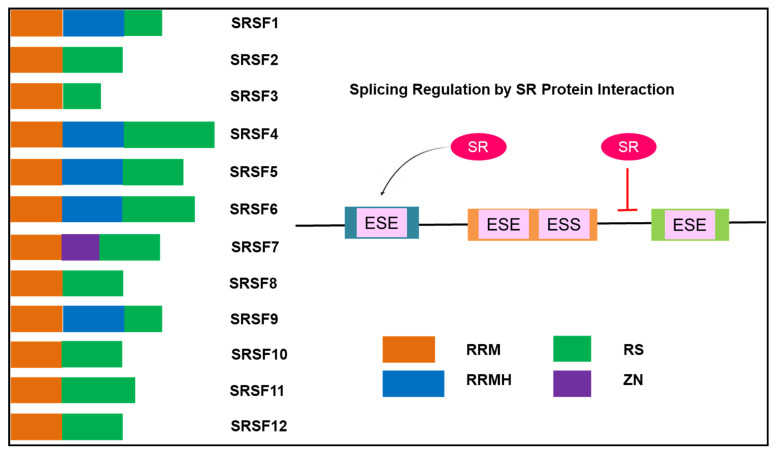
Alternative splicing regulation by SR binding. The domain structure of the 12 serine-rich (SR) proteins indicating the RRM (RNA recognition motif), RRMH (RRM homology), RS (arginine/serine-rich domain), and Zn (Zink knuckle). SR proteins bind to exonic splicing enhancers (ESEs), facilitating splice site recognition and stimulating the activation of splicing. In comparison, splicing is inhibited by the binding of the SR to introns [64,65,66].

The spliceosome is crucial in regulating alternative splicing. In addition, other regulatory factors that are short DNA sequences, known as exonic splicing enhancers (ESEs), exonic splicing silencers (ESSs), intronic splicing enhancers (ISEs), and intronic splicing silencers (ISSs), ensure accurate splicing [60]. The splicing regulators have either a positive or negative effect on alternative splicing. ESE and ISE are cis-acting elements that are capable of binding the SR protein family to facilitate recognition of exons and initiate assembly of the spliceosome prior to alternative splicing (Figure 3) [61]. SR proteins are also known as SR splicing factors (SRSF) with SRSF1–12 as the major proteins in this family that have been identified as splicing regulators [62]. SR proteins have other vital cellular functions that are hallmarks of cancer, namely, cell cycle regulation, apoptosis, genome stability, cell adhesion and metastasis [27,63], and angiogenesis [64].

**Figure 4:**
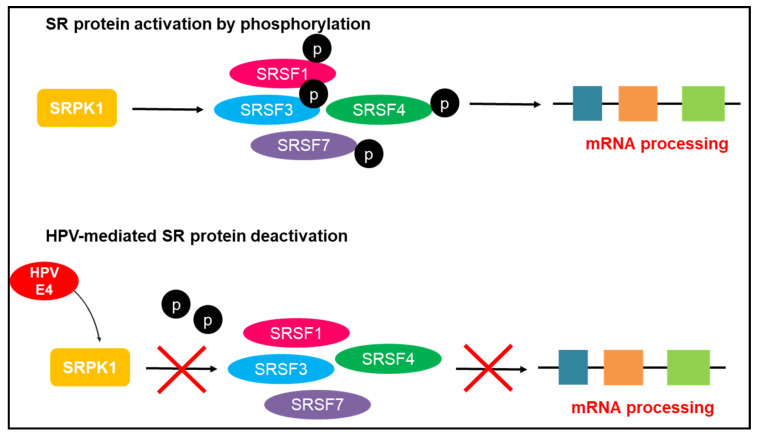
Human papillomavirus (HPV)-mediated disruption of accurate mRNA processing. The binding of HPV E4 protein leads to the deactivation of SR splicing factors (SRSFs) 1, 3, 4, and 7 by the loss of phosphorylation. This concomitantly inhibits pre-mRNA splicing, leading to inaccurate splicing and production of oncoproteins that give rise to malignancy [18,67]. SRPK: SR protein-specific kinase.

The SRSF regulates splicing by determining the cycle of phosphorylation of SR proteins. CDC-like kinases (Clks), SR protein-specific kinases (SRPKs), and Topoisomerase 1 modulate the activation of SRSFs through a cycle of phosphorylation and dephosphorylation [18]. In the event of dephosphorylation, SRSFs begin to accumulate in the cytoplasm [64]. In comparison, phosphorylated SRSFs are transported to the nucleus to stimulate splicing. The SRPKs are capable of splicing regulation by the binding action to Clks in the nucleus and the cytoplasm [21]. In addition to splicing regulation, evidence suggests that SRPKs are able to modulate viral genomic material such as the HPV [18,21,31]. Evidence shows the binding of HPV E4 protein to SRPK1 [18]. This binding action impedes activation of SR protein by inhibiting the phosphorylation of SRSF1, SRSF3, SRSF4, and SRSF7 and impedes the pre-mRNA processing (Figure 4) [67]. This leads to aberrant cellular splicing that results in oncoprotein production and cervical cancer [18].

Evidence sheds light on the oncogenic role of SRSF1 [68] and a recent report shows its involvement in cervical malignancy [21]. Mole et al. (2020) showed enhanced levels of SRSF1 in cervical cancer cells. The authors showed the trans-activation of the SRSF1 gene promoter by the high-risk HPV16 E2 protein, with differing levels in the nucleus and cytoplasm [21]. Modifications of SRSF1 abrogate alternative splicing and facilitate genomic instability and cervical malignancy. Henceforth, the results suggest that the increased cytoplasmic levels of SRSF1 are associated with early tumour progression [21]. Other evidence shows the interaction of SRSF1 binding to long non-coding RNAs (lnRNA) to regulate expression levels of keratin 17. Cervical cancer cells display enhanced levels of keratin 17. Dong et al. (2019) showed the interplay between SRSF1 and lnRNA to modulate expression of keratin 17 through alternative splicing in cervical cancer [22]. In addition to SRSF1, SRSF3 regulates the expression of a number of genes and the overexpression of SRSF3 has been shown to modulate cell proliferation by inducing G2/M cell cycle arrest and apoptosis [69,70]. SRSF3 induces production of interleukin enhancer binding factor 3 (ILF3) isoform 1 and 2 through aberrant alternative splicing. These isoforms are involved in malignant transformation [71]. Furthermore, SRSF3 regulates expression of p300, a tumour suppressor, and induces cell proliferation [70]. In HPV-infected cervical cells, SRSF3 plays a significant part in the E6* splicing that is vital for E7 production [72] and in E1^E4 for viral replication [73]. Silencing SRSF3 in HPV-infected cells shows downregulation of viral E6 and E7 [72] and suppresses the E1^E4 splicing [73]. These results highlight the oncogenic potential of SRSF3 that may lead to cellular transformation and may contribute to cervical cancer [69].

DNA damage response plays a vital role in maintaining genomic stability and preventing carcinogenesis. Several important genes are involved in DNA damage pathways such as RAD51, ATM, p53, and ERCC1 [74]. Detecting modifications in DNA repair genes could be beneficial as biomarkers for diagnosis, prognosis, and targets for therapy. For instance, evidence shows the upregulated RAD51 mRNA levels in cervical cancer, which are associated with poor prognosis [75]. In addition to somatic mutations, HPV induces DNA damage in cervical cancer cells [76] and the resulting DNA damage response gene expression serves as prognostic biomarkers [77]. New evidence shows the association between SRSF6 and DNA damage genes. Yang et al. (2020) evaluated the function of SRSF6 in cervical cancer cells and showed that overexpressed SRSF6 influenced the alternative splicing of DNA damage genes [78]. SRSF6-induced aberrant alternative splicing of DNA damage genes is associated with the hallmarks of cancer such as cell proliferation, tumour progression, and apoptosis [78]. Elucidating the functional impact of SRSF6 in alternative splicing of DNA damage genes could offer a target for cervical cancer therapy.

### 2.2. HPV-Mediated Disruptions in Heterogeneous Ribonucleoproteins (hnRNPs)

**Figure 5:**
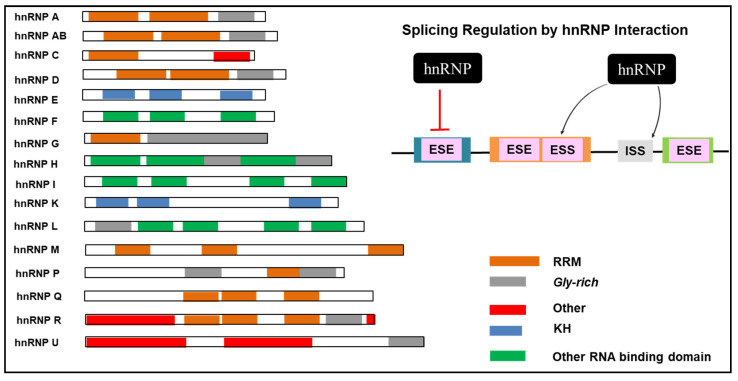
Alternative splicing regulation and the structural domains of hnRNP family. The domain structure of the hnRNP showing the RRM (RNA recognition motif), KH (K homology domain), and other RNA binding domain that is structurally different from RRM. hnRNP negatively regulates this process by binding to either exonic splicing silencers (ESSs) or intronic splicing silencers (ISSs). In addition, hnRNP blocks the activity of exonic splicing enhancers (ESEs) by binding to it [34,65,80].

The ESS and ISS act as negative regulators to repress alternative splicing and bind to the hnRNP family of proteins. Similar to SR proteins, the hnRNPs can either positively or negatively regulate splicing by binding to ESS and ISS, negatively prompting exon definition (Figure 5). There are currently at least 20 hnRNPs identified with several important cellular functions including alternative splicing [31]. Loss of regulation in hnRNPs leads to modified gene expression of tumour suppressors and other cancer-related genes [27,79]. Henceforth, hnRNPs are implicated in malignant transformation and could be scrutinised as potential cancer-related biomarkers.

Alternative splicing events are frequent in cervical cancer and are significantly associated with diagnosis and prognosis. Major splicing factors promote cervical malignancy by facilitating the production of HPV mRNAs and oncoproteins required. In addition, cellular oncogenic protein production is favoured to enhance the development of cervical cancer (Table 2). Cervical cancer cells have elevated expression of hnRNPs. For instance, hnRNPA1 is highly expressed in cervical cancer cells and can disrupt cancer-related genes. The alternative splicing of pyruvate kinase mRNA is induced by hnRNPA1 and favours aerobic glycolysis, resulting in uncontrolled cell proliferation. In the event where hnRNPA1 is downregulated, cancer-specific apoptosis is induced. hnRNPA1 is thus a good biomarker for cervical cancer diagnosis [23]. Another recent study investigated prognostic biomarkers of alternative splicing in cervical cancer and revealed hnRNPA1, ubiquitin C, and RNA polymerase II subunit L as effective prognostic biomarkers [81]. As a crucial component in alternative splicing, scrutinising the aberrant splicing induced by hnRNPA1 in cervical cancer is critical. Additionally, during the HPV infection-related differentiation of cervical epithelial cells, hnRNPA1 is further upregulated and enables oncoviral protein transcription. Deleterious mutations in hnRNPA1 have been identified and may alter expression levels contributing to aberrant alternative splicing, mRNA processing, and translation [82].

Prolonged HPV infections influence cellular and viral alternative splicing to enhance viral oncogene production, leading to malignant transformation of the cervix. Malignant transformation is initiated and sustained by the high-risk HPV16 E6 and E7 proteins that interact with tumour suppressor genes p53 and retinoblastoma protein (pRb), respectively. The interaction of E6 with p53 results in apoptosis, whereas E7 steers cell proliferation by interacting with pRb [83,84]. Moreover, E6 and E7 are essential in viral replication [85]. Zheng et al. (2020) showed splicing regulation of E6 and E7 by cellular hnRNPA1 and hnRNPA2 [20]. This study revealed the direct interaction of hnRNPA1 and hnRNPA2 with high-risk HPV16 splice site SA742 and SA409. The authors showed the inhibition of SA409 when hnRNPA1 is overexpressed and favouring viral E6 mRNA production. In comparison, when hnRNPA2 is upregulated, the viral E6Ê7, E1, and E4 mRNA transcripts are favoured [20]. Adequate amounts of both E6 and E7 transcripts are required for the development of cervix carcinoma. Furthermore, evidence also shows that HPV interacts with hnRNPA1 and the silencing of hnRNPA1 suppresses E6 intron retention [73]. Hence, targeting hnRNPA1 and hnRNPA2 to modulate viral E6 and E7 mRNA transcripts may provide novel therapeutic strategies.

## 3. Alternative Splicing and Therapy Resistance

**Figure 6:**
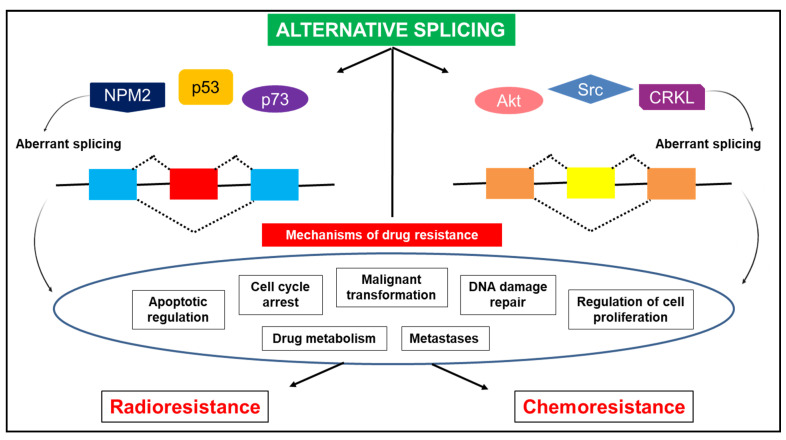
Alternative splicing-induced drug resistance. Aberrant splicing events of vital genes in cervical cancer cells promote drug resistance through several mechanisms by regulating apoptosis, cell cycle arrest, cell proliferation, and DNA damage response. In addition, splice variants may also alter drug targets that effect drug metabolism and lead to chemoresistance and alter the sensitivity to radiotherapy [86,87,89,95,97,98,99].

Drug resistance is a considerable hurdle in cancer treatment and management. Aberrant alternative splicing events are a common theme in cancer drug resistance and, therefore, strategies targeted to silence variants that promote drug resistance are highly warranted. Aberrant splice variants can promote resistance to chemotherapy and radiotherapy [24,86,87,88] by mechanisms that include apoptotic regulation, modified drug metabolism, response to DNA damage, and regulation of cell proliferation (Figure 6) [89]. Radiotherapy is an important therapeutic modality for the management of advanced cervical cancer and radioresistance may be detrimental. In cervical cancer, a splice variant of nucleophosmin (NPM) protein resulting from alternative splicing causes radioresistance [86]. NPM functions in mRNA processing, genome stability, and apoptotic regulation [90]. Enhanced expression of the NPM2 variant is correlated with a radio-protective function. Evidence shows that silencing the NPM2 splice variant decreases radioresistance in cervical cancer cells in a dose-dependent manner [86]. Similarly, enhanced levels of ΔNp73, a splice variant of p73, have anti-apoptotic functions and display radioresistance in cervical cancer cells [91]. p73 (i) is a p53 homologue that expresses the oncogenic isoform ΔNp73 [92]; (ii) functions in DNA damage repair, cell cycle regulation, and apoptosis with p73 polymorphism closely associated with cervical cancer [93]; and (iii) is a prognostic biomarker for cervical cancer [94]. Cervical cancer cells exposed to high-LET radiation degrade ΔNp73 to exhibit enhanced apoptosis and cell cycle arrest at the G2/M phase when compared with low-LET radiation [91]. In addition, ΔNp73 promotes malignant transformation by interacting with RAS and inducing drug resistance to chemotherapy and radiotherapy [87]. Furthermore, the HPV oncoprotein, E6, suppresses the activity of p53 expression and alters sensitivity to radiotherapy. The overexpression of the splice variant, p73α, in p53 deficient cervical cancer cells, enhances sensitivity to radiotherapy [95]. These results highlight the importance of targeting aberrant splice variants to reverse radioresistance in cervical cancer, which is significantly relevant in treating advanced metastatic disease.

Cervical cancer is often managed with chemotherapy and radiotherapy concurrently. An estimated 50% of patients do not attain a complete response to therapy due to resistance. Alterations in molecular pathways that promote drug resistance are potential drug targets to counteract resistance [96]. For instance, the CRK-like (CRKL) adapter protein is overexpressed in approximately 50% of cervical cancers. Moreover, evidence shows that CRKL significantly regulates alternative splicing of pre-mRNA in cancer-related genes in cervical carcinoma to promote malignant transformation, metastases, and chemoresistance by binding to BCR-ABL and activating the Src and Akt signally pathway through phosphorylation [97,98]. Additionally, recent evidence shows the role of AKT3 mRNA in inducing cisplatin resistance [99]. By blocking the activity of Src and Akt through pharmacological inhibitors such as dasatinib [97] and fucoxanthin [100], respectively, aberrant splicing events that facilitate chemoresistance in cervical cancer can be reversed and promote a complete therapy response in advanced metastatic disease.

## 4. Clinical Utility of Biomarkers in Cervical Cancer

**Figure 7:**
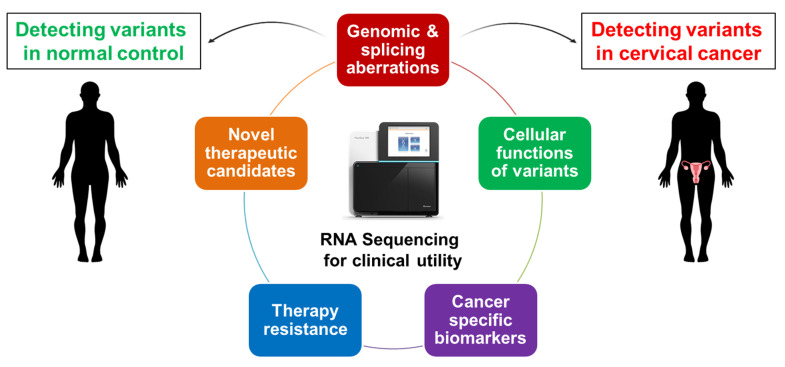
Overview of clinical biomarker identification. Aberrant alternative splice variants are often expressed in significantly higher levels compared with normal splice variants that can be identified through next generation sequencing (NGS). These aberrations can contribute to the development of tumourigenesis, therapy resistance, and poor prognosis. The effects of aberrant alternative splicing can be addressed by identifying cervical cancer-specific genomic and splicing aberrations that are clinically relevant for novel diagnostic, prognostic, and therapeutic purposes [24,81].

Altered expression of splicing regulators, deleterious mutations in splicing regulators and splicing regulatory sequences, and suppressed activity of splicing regulators can cause aberrant alternative splicing, which may result in tumourigenesis and therapy resistance (Figure 7). However, alternative splicing biomarkers have been studied extensively as potential targets of novel therapy [24,27]. The current diagnostic and prognostic indicators of cervical cancer are largely clinicopathology and HPV screening intensive. With the introduction of NGS, large-scale RNA sequencing can be clinically utilised to identify tissue-specific molecular biomarkers. Subsequent to the identification of onco-biomarkers, functional biological assays are imperative to characterise the properties of effective and clinically significant biomarkers for novel clinical utility in diagnosis, prognosis, and therapeutic interventions [27].

Reversing aberrant alternative splicing or silencing oncogenic variants could offer therapeutic strategies in managing cervical cancer. Pharmacological agents are frequently evaluated for their splicing inhibitory or silencing effects in cancer cells. The current alternative splicing modulators studied are small molecule splicing inhibitors, transsplicing, antisense oligonucleotides, and gene therapy. These modulators can regulate alternative splicing by controlling the functioning of spliceosomal activity [27]. For instance, caffeine suppresses the expression of SRSF2/3 and p53α, while upregulating the alternative spliced variant of p53β. Caffeine regulates cellular functions such as cell cycle arrest, DNA damage, and apoptosis by modulating the SRSF3 [101,102]. Cervical cancer cells treated with caffeine showed tumour suppression through the modulation of splicing factors. In addition, the recent evidence shows that pladienolide B inhibits the splicing factor SF3b1, which is a subunit of the spliceosome, to induce the G2/M cell cycle arrest, apoptosis, and p73 splicing in cervical cancer cells [103]. Other small molecules evaluated in cervical cancer include RI-1, a RAD51 inhibitor [104]. Modified gene expression is a central characteristic of cancer cells such as the altered expression of RAD51 mRNA in cervical cancer cells compared with healthy cells [75]. RI-1 promotes cell cycle arrest from G0/G1 to S phase and inhibits the RAD51-induced cell proliferation in cervical cancer cells [104]. These results indicate the potential of pharmacological agents to regulate alternative splicing in cervical cancer and their therapeutic potential.

Inhibiting splicing factors can evoke a tumour suppressive function. For instance, blocking the function of SRSF1 may contribute to apoptotic activity. Cervical cancer cells treated with an AURKA kinase inhibitor, such as the pharmacological agent VX-680, downregulate the post-transcriptional expression levels of SRSF1 [105]. AURKA kinases, part of the aurora family of proteins, are cell division regulators. Dysregulation of these proteins leads to uncontrolled cell division and proliferation, resulting in malignancy [42]. Cervical cancer cells treated with VX-680 promote aberrant alternative splicing of apoptotic regulating genes, Bcl-x and Mcl-1, and inhibit the anti-apoptotic function of SRSF1, leading to apoptosis [105]. Silencing of SRSF1, therefore, signifies a novel therapeutic target for cervical cancer.

## 5. Conclusions

The mortality associated with cervical cancer is increasing at an alarming rate. The development of cervical cancer is largely influenced by HPV infections in low- and middle-income countries that add to this encumbrance. Vaccination programs addressing HPV have been successful in lowering HPV infections in high-risk women. Moreover, screening and prevention programs are useful in early detection and treatment. In addition to HPV infections, molecular alterations at the RNA level contribute to cervical carcinoma. These include modifications in cellular alternative splicing induced by HPV. RBPs like SRs and hnRNPs are essential in maintaining the stability and packing of mRNAs, as well as transport to the cytoplasm for further processing. These processes are intricately balanced by several splicing factors and proteins to ensure accurate alternative splicing. Despite the stringent regulation, SR proteins and hnRNPs are often dysregulated in cervical cancer and lead to aberrant alternative splicing of many important cancer-related genes, including therapy resistance. For these reasons, SR proteins and hnRNPs are ideal candidates for drug targets. Hence, identifying biomarkers crucial to the development of cervical malignancy, its pathogenesis, and splice variants that are highly expressed in cervical cancer will be beneficial in developing novel therapeutic targets, especially in low- and middle-income countries where the burden of cervical cancer is rapidly increasing.

## Figures and Tables

**Table 1:** Overview of biomarkers associated with cervical cancer.

Biomarkers	Biological Function	Modifications in Cervical Cancer	Clinical Utility	Ref.
HPV E6	p53 degradation	Overexpressed in cervical cancer cells	Screening and prevention	[38,39]
HPV E7	pRb degradation	Overexpressed in cervical cancer cells	Screening and prevention	[38,39]
AURKA	Genomic stability	Overexpressed in precancerous and cancerous cervical cells	Early detection	[40,41,42]
DTL	Checkpoint regulation	Overexpressed in cervical cancer cells	Early detection	[41]
HMGB3	Maintain balance in stem cell population	Overexpressed in cervical cancer cells	Early detection	[41]
KIF2C	Cell proliferation	Overexpressed in cervical cancer cells	Early detection	[41]
NEK2	Mitotic and cell cycle regulation	Overexpressed in cervical cancer cells	Early detection	[41]
RFC4	DNA replication	Overexpressed in cervical cancer cells	Early detection	[41]
p16ink4a	Tumour suppressor	Overexpressed in precancerous and cancerous cervical cells	Screening and diagnosis	[43,44,45]
Ki-67	Cell proliferation	Increased expression in proliferating epithelial cervical lesions	Screening and diagnosis	[43,46]
MCM2/TOP2A	DNA synthesis	Overexpressed in cervical dysplasia	Diagnosis	[38,47]
MSI1	RNA binding protein	Overexpressed in cervical cancer cells	Diagnostic and therapeutic	[48,49,50,51]
miR-21, miR-127 and miR-199a	-	Increased expression in cervical cancer cells	Prognosis	[39]
miR-143, miR214, miR-218 and miR-34a	-	Decreased expression in cervical cancer cells	Prognosis	[39]
ALDH1	Cellular differentiation and proliferation	Overexpressed in cervical cancer cells	Prognosis and predictive	[48,51,52]
EGFR	Transmembrane protein	Overexpressed in cervical cancer cells	Prognosis and predictive	[53,54]
Oct3/4	Transcription factor	Overexpressed in cervical cancer cells	Prognosis and predictive	[48,52,55]
Sox2	Transcription factor	Overexpressed in cervical cancer cells	Prognosis and predictive	[51,55]
CD49f	Stem cell marker	Overexpressed in cervical cancer cells	Prognosis and predictive	[48,51,56]
CD133	Cell surface antigen	Overexpressed in cervical cancer cells	Prognosis and predictive	[48,57]
CD44	Cellular differentiation and proliferation	Overexpressed in cervical cancer cells	Predictive	[48,58]
KAT2B	Mitotic and cell cycle regulation	Downregulated in cervical cancer cells	Predictive	[59]

**Table 2:** The role of major splicing factors, the human papillomavirus (HPV) binding region, and function of transcripts in cancer progression.

Splicing Factor	HPV Binding Region	HPV16 mRNA	Cancer Promoting Function
SRSF1	E4	Production of E6/E7 mRNA	Apoptotic regulation
SRSF3	E4	Production of E6/E7 mRNA	Increased cell proliferation
SRSF9	E4	Production of late mRNAs	Increased cell proliferation and suppressed apoptosis
hnRNPA1	L1	Production of the isoform E6*I/E7	Apoptotic regulation
hnRNPA2/B1	E4	Production of the isoform E6*I/E7	Apoptotic regulation
hnRNPC	Early 3′-UTR	Production of L1 mRNA	-
hnRNPD	E4	Production of late mRNAs	-
hnRNPE1/E2	L2	Inhibition of L2 mRNA	-
hnRNPG	E4	Production of late mRNAs	-
hnRNPH	L2	Inhibition of late mRNAs	-
hnRNPI	Early 3′-UTR	Inhibition of late mRNAs	Cell proliferation and cell invasion
hnRNPK	L2	Inhibition of L2 mRNA	Cell cycle regulation
hnRNPL	E4 and L1	Inhibition of late mRNAs	-

UTR: Untranslated region. Reviewed in [31].

## Data Availability

Not applicable.
